# Breathing Fresh Air in the City: Implementing Avenue Trees as a Sustainable Solution to Reduce Particulate Pollution in Urban Agglomerations

**DOI:** 10.3390/plants12071545

**Published:** 2023-04-03

**Authors:** Mamun Mandal, Robert Popek, Arkadiusz Przybysz, Anamika Roy, Sujit Das, Abhijit Sarkar

**Affiliations:** 1 Laboratory of Applied Stress Biology, Department of Botany, University of Gour Banga, Malda 732103, West Bengal, India; 2 Section of Basic Research in Horticulture, Department of Plant Protection, Institute of Horticultural Sciences, Warsaw University of Life Sciences-SGGW (WULS-SGGW), Nowoursynowska 159, 02-776 Warsaw, Poland

**Keywords:** particulate pollution, urbanization, green infrastructures, avenue trees

## Abstract

The issue of air pollution from particulate matter (PM) is getting worse as more and more people move into urban areas around the globe. Due to the complexity and diversity of pollution sources, it has long been hard to rely on source control techniques to manage this issue. Due to the fact that urban trees may provide a variety of ecosystem services, there is an urgent need to investigate alternative strategies for dramatically improving air quality. PM has always been a significant concern due to its adverse effects on humans and the entire ecosystem. The severity of this issue has risen in the current global environmental context. Numerous studies on respiratory and other human disorders have revealed a statistical relationship between human exposure to outdoor levels of particles or dust and harmful health effects. These risks are undeniably close to industrial areas where these airborne, inhalable particles are produced. The combined and individual effects of the particle and gaseous contaminants on plants’ general physiology can be detrimental. According to research, plant leaves, the primary receptors of PM pollution, can function as biological filters to remove significant amounts of particles from the atmosphere of urban areas. This study showed that vegetation could provide a promising green infrastructure (GI) for better air quality through the canopy and leaf-level processes, going beyond its traditional role as a passive target and sink for air pollutants. Opportunities exist for urban GI as a natural remedy for urban pollution caused by PMs.

## 1. Introduction

Due to rapid urbanization, more people live in cities and are exposed to harmful air pollution [1]. As a result, today’s population is affected by air pollution, which is a severe health concern [2]. Air pollution has a wide range of chemical compositions depending on the rate of emission, source, and weather factors such as wind and sunlight. Nitric oxide (NO), nitrogen dioxide (NO_2_), sulphur dioxide (SO_2_), ozone (O_3_), and carbon monoxide (CO) are some of the gaseous pollutants in the air [3]. Particulate matter (PM) components of air pollution contain carbon-reached particles; it is a complex combination of various substances made up of microscopic particles, liquid droplets, metals, organic compounds, and dust or soil particles [4]. Sulfates, nitrates, polycyclic aromatic hydrocarbons (PAHs), endotoxin, and metals, including copper, iron, zinc, nickel, and vanadium, are typical PM constituents [5]. Based on particle size, PM is divided into three categories: coarse (size <10 μm), fine (size <2.5 μm), and ultrafine (size <0.1 μm) [6].

**Figure 1:**
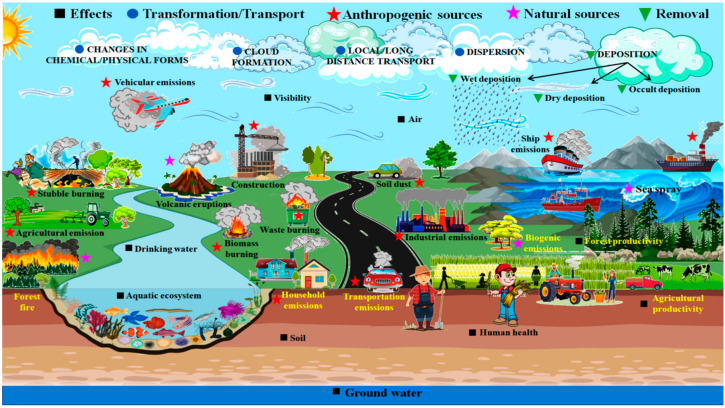
A diagrammatical representation showing the different sources of PM pollution (natural and anthropogenic) that can affect different ecosystems. PM can change chemical and physical forms and form a cloud in the air, and can be dispersed and transported long/local distances. PM is deposited (wet, dry, occult) in the environment by three different types of processes.

PM particles originate from several natural and anthropogenic sources, which are represented in detail in Figure 1. However, the organic and metal content of particles varies by region. Fine and ultrafine particles have more detrimental health effects [7] than coarse particles because coarse particles do not pass beyond the upper bronchus [3], but fine and ultrafine particles penetrate the small airways and alveoli [4] and potentially enter the bloodstream [8]. Increased PM_2.5_ levels [3] strongly correlate with negative health impacts, primarily on the respiratory and cardiovascular systems. Premature mortality, allergies, and even lung cancer and premature death are all side effects of long-term or chronic exposure to PM [5,9]. Additionally, PM pollution affects ecosystems, including agriculture and the climate, and has a significant financial cost [10].

Several steps have been taken to reduce particle air pollution at the source to control atmospheric concentration levels due to the harmful health impacts, including emission reductions, restrictions, and objectives (e.g., WHO Air Quality Guidelines) [11]. Different air filtering techniques have been adopted to improve indoor air quality [12]. Air filtration techniques may successfully reduce the deterioration of indoor air quality and remove air contaminants, but they are quite costly and relatively local [13]. Therefore, vegetation is the only efficient way to remove it. One of these attempts involves investigating the possible mitigating impact of vegetation [14]. Compared to other terrestrial surfaces, vegetation is more successful at capturing gases, particles, and aerosols from the atmosphere (by depositing them on its leaves and stems) [15]. As a result, plant surfaces can act as both a sink for air particles and a point of absorption for some elemental nutrients attached to the PM [16]. The application of urban green infrastructure (GI) is a possible natural remedy.

For the majority of atmospheric contaminants, trees/shrubs are effective sinks [15]. According to an assessment, Guangzhou’s urban vegetation can remove 312 Mg of SO_2_, NO_2_, and total suspended particles (TSP) annually [17]. In the United States, urban trees have been calculated to remove 711,000 tons (t) of PM per year (y) [18]. In Chicago, existing urban woods are thought to remove 212 t of PM_10_ annually [19]. Furthermore, according to model studies, urban trees reduce 0.2–1.0% of PM_10_ emissions [18]. In Strasbourg, France, Selmi et al. [20] conducted the first study to use the i-Tree Eco model to quantify the removal of air pollutants by urban trees. The study reported that the trees managed by the city could remove 5 t of PM_2.5_, 12 t of PM_10_, 14 t of NO_2_, 1 t of CO, 56 t of O_3_, and 1 t of SO_2_ pollutants annually. The urban canopy of the Greater London Authority (GLA) was estimated to remove PM_10_ of between 852 and 2121 t yearly using the Urban Forest Effects (UFORE) model [21]. Wu et al. [22] reported that urban vegetation in Shenzhen City, China removed 1000.1 t of PM_2.5_, and the average removal rate was 1.6 g m^−2^ per year.

PM deposition in the air is influenced by precipitation and wind, and PM concentration is also affected [23]. The dispersion of PM in the air is caused by turbulence brought on by higher wind speeds. In contrast, the geographical distribution of PM and the direction of its transport are influenced by wind direction [23]. Precipitation is one of the leading natural mechanisms that lower the amount of PM in the surrounding air. Its scavenging effects come from wet deposition onto surfaces, such as vegetation, and wet removal from the atmosphere [24]. According to Popek et al. [23], the precise way in which wind and precipitation affect PM concentration depends on particle size. While PM_2.5-10_ concentration rose because of resuspension in high wind, PM_0.2–2.5_ concentration steadily dropped with increasing wind speed. On coarse PM, precipitation had a purifying effectiveness that was more than twice as high as that on fine PM. It is also crucial to remember that other climatic factors, such as air humidity, temperature, and vertical inversions in the lower troposphere will impact PM concentration in the atmosphere [24]. The selection of species is another factor that must be considered. The features of various species, including leaf size, stomata, vegetation structure, and leaf microstructure, will impact the effectiveness of capture [13]. Particles traveling in an air stream that bends around an object (e.g., leaf or stem) are propelled onto the object by their inertia, pushing them past the boundary layer. For instance, trees are more successful at absorbing these chemicals than shorter vegetation due to their enormous canopy surface area of leaves, stems, and branches and the air turbulence caused by their structure [25]. It was discovered in the West Midlands of England that forests gathered three times as much PM_10_ as grassland [26].

This review focused on how urbanization and contemporary civilization cause PM pollution in urban areas, with implications for people and all ecosystems. Finally, the study shows that the “Tree Avenue” method of reducing PM pollution can act as a biological filter that effectively removes large amounts of PM from urban air. This approach may prove to be a cost-effective technology and improve the aesthetic significance of urban agglomerations.

## 2. Urbanization: The Fate of Modern Civilization

Urbanization is a process that causes cities to expand owing to industrialization and economic development. That causes changes in specialization, labor division, and human behaviors specific to urban environments. Urbanization arises due to the expansion in the density and extent of urban regions [27]. The world’s population is growing, especially in cities, where more than 60% of people are expected to dwell by 2050, making this the “Urban Century” [28,29]. Globally, unchecked urbanization has accelerated environmental deterioration, which has led to several issues, including housing scarcity, deteriorating water quality, noise, and heat, as well as issues with the disposal of hazardous solid waste [30]. Reyna-Bensusan et al. [31] reported that, in the Municipality of Huejutla, Mexico, about 24% of the total solid waste produced was burned. It has been estimated that approximately 8882 t of waste are burned annually, generating 1.97 kg black carbon (BC) t^−1^, 9.8 PM_2.5_ t^−1^ and 11.9 kg PM_10_ t^−1^, which significantly contributed to 17.5 t BC y^−1^ (38,553 t CO_2_-equivalent per year), 87.0 t PM_2.5_ y^−1^, and 105.7 t PM_10_ y^−1^ for a total of 313.7 kg CO_2_-equivalent y^−1^ per capita.

### 2.1. The Rural–Urban Shift

Urbanization is moving from rural to urban lifestyles, which results in a rise in the percentage of people living in urban areas [32]. The United Nations reported that urbanization has increased about tenfold since the 1950s and, today, 55% of the world’s population lives in urban areas, up from 30% in 1950. The percentage is expected to rise to 60% in 2030 [33] and 66% in 2050 [34]. Africa and Asia will account for almost 90% of this rise. The socioeconomic situations of individuals are often bettered by urbanization, which also increases access to social services, literacy, education, and health. Urbanization also promotes economic expansion [35]. Rural–urban interaction results from the extension of urban land onto the rural–urban fringe, supported by urban expansion [32,36].

The rate of global urbanization conceals significant regional variations in the degree of urbanization. North America has the highest urbanization rate, with 82% of its people living in urban areas. Africa continues to be predominantly rural, with 43% of its inhabitants residing in urban regions, whereas Asia had around 50% urban regions in 2018 [37]. Over half of the world’s population lived in urban areas in 2014. More than two-thirds of people worldwide lived in rural areas in 1950, while less than one-third of people resided in urban areas [37,38]. The world’s urban population increased more than four times between 1950 and 2018, from a projected 0.8 billion to 4.2 billion [39]. Over the following 35 years, this distribution is estimated to move even more in favor of urban regions; the world population will be two-thirds urban and one-third rural by 2050, roughly the reverse of the condition in the middle of the 20th century [38]. In 2018, more than half of the people in Northern America lived in cities with 500,000 or more residents. Latin America and the Caribbean is the area with the most significant proportion of inhabitants concentrated in large cities; in 2018, of the area’s entire population, 14.2% lived in the six megacities with more than10 million people [40]. In 2018, more than half of the population resided in rural areas in Africa and Asia, a decreasing percentage on both continents. Cities with 500,000 or more residents are anticipated to increase by 57% in Africa and by 23% in Asia between 2018 and 2030. The population of Delhi, India, is expected to rise by more than 10 million people between 2018 and 2030 [40]. Urbanization in the developing world is progressing much faster (about 4% a year) than in developed countries [36].

### 2.2. Impacts of Urbanization on PM Generation and Concentrations

The processes of industrialization and urbanization both serve as a framework for socioeconomic growth [41]. According to [42], well-managed urban expansion and development may benefit rural lives by generating chances for non-farm employment, access to sophisticated extension services, and a strong market for agricultural goods [43]. Contrarily, unplanned urbanization harms rural livelihoods [36] because it alters land use and cropping patterns, reduces the amount of arable land available, increases unemployment farmer numbers, raises the cost of food commodities, results in scarce and poor-quality water, and puts more pressure on the competition between residential and agricultural uses of natural resources [44]. Urbanization expands built-up regions, increasing rural poverty [36]. For example, according to a survey, the rural regions of Mumbai’s metropolitan area’s 34 km^2^ (17.8%) forest lands have been developed [45]. Peripheral rural regions combine elements of the urban and rural worlds and see several changes [46].

However, such procedures significantly raise the danger of harm from environmental contamination, ecological disturbances, hydrometeorological catastrophes, and exacerbated climate change [47]. It is crucial to remember that most of Asia’s metropolitan areas are situated along the Indian and West Pacific Ocean’s coast. The probability of cyclones, coastal floods, urban floods, sea level increases, etc., has significantly risen due to tsunamis and climate change [48]. The World Economic Forum (WEF) predicts that, by 2050, city inhabitants’ exposure to different dangers, such as earthquakes, urban floods, cyclones, air pollution, water pollution, and storm surges, will have increased by a factor of two [41].

The disparate degrees of social-economic development worldwide can be partly blamed for the inconsistent study findings. In this context, Northam’s [49] S-curve theory of urbanization may offer a theoretical base for thoroughly comprehending the relationship between urbanization and concentrations of PM_2.5_ in various places at various developmental phases. The theory states that when the proportion of people living in cities rises, urbanization progresses through three separate stages: the beginning stage (urban population share ≤ 30%), the acceleration stage (30% < share of urban population < 70%), and the terminal stage (urban population share ≥ 70%). Additionally, at various phases of urbanization, there are considerable differences in the size and rate of population movement, industrial structure, urban area growth, and residential patterns, all of which are directly related to energy usage and air pollution emissions [50]. For example, in the beginning stage, the majorities of people in an area tend to be equally dispersed and are primarily occupied with fishing and agriculture. The economy and urbanization are still in the early development stages, and solid biomass combustion is the primary source of air pollution issues. At this stage, air pollution is not a significant concern. However, when an area moves into the acceleration stage of urbanization, industry, mining, and the rate of rural–urban movement all increase, which increases the usage of fossil fuels and air pollution emissions [51]. In the last stages, rising environmental awareness, improvements in pollution control and renewable energy technology, and stringent regulatory laws will result in a substantial reduction in pollutant emissions [52]. These theoretical and empirical claims, in short, imply that various cities and developmental phases should experience urbanization’s environmental effects differently. According to earlier research, urbanization significantly influences air pollution in metropolitan areas at various economic growth stages (such as in underdeveloped, developing, and developed countries).

**Figure 2:**
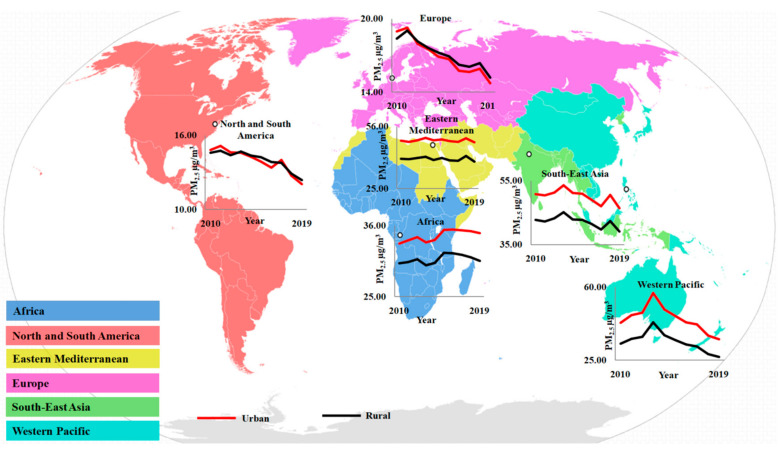
Graphical representation showed the PM_2.5_ concentration level in urban and rural areas from 2010 to 2019 (https://www.who.int/data/gho/data/themes/air-pollution/who-air-quality-database, accessed on 11 November 2022). The representation shows that the rural area level of concentration is lower compared to that in the urban area in most cases.

*Urbanization* has been identified as a substantial contributor to PM_2.5_ emissions, among other sources, according to Wang et al. [53]. They found that underdeveloped countries have a considerably larger positive response ofPM_2.5_ to rising transport-related emissions and urbanization than developed and developing countries (Figure 2).

#### 2.2.1. In Developed Countries

In developed countries, urbanization has a reducing influence on PM_2.5_ concentrations. Between 1998 and 2014, the average percentage of urban residents in developed countries rose from 76.03 to 78.9 percent; on the other hand, concentrations of PM_2.5_ fell from 15.27 μg/m^3^ to 14.91 μg/m^3^ [53]. In addition, urban sprawl has typically resulted from urbanization in most developed countries, leading to increasing proportions of people living in cities but lower urban population densities [53].

#### 2.2.2. In Developing and Under Developed Countries

The recent decades’ rapid urbanization in developing nations has significantly exacerbated the rise in concentrations of PM_2.5_. Between 1998 and 2014, the average concentration of PM_2.5_ in developing countries rose from 19.91 μg/m^3^ to 21.57 μg/m^3^ [53]. Kinney et al. [54] noted that while there is limited data from a larger sample of developing nations, motor vehicle traffic is a significant PM pollution source in Kenya. Han et al. [55,56] and Guan et al. [57] reported that, in Chinese cities, urbanization significantly influenced the rise in PM_2.5_ concentrations, according to a quantitative analysis of the geographical patterns of PM_2.5_ concentrations. According to Wang et al. [51], there is a direct association between PM_2.5_ concentrations (ranging between 18.7 and 131.4 μg/m^3^) and urban area size, population density, the proportion of the secondary industry, and urban population. Some studies also investigated differences in the link between urbanization and PM_2.5_ emissions, extending the research’s scope beyond the national to the international level. Han et al. [58,59] discovered, for instance, that while PM_2.5_ concentrations in major cities in North America, Latin America, and Europe showed little variation or only a slight increase, those same cities’ PM_2.5_ concentrations in India and Africa showed a “U” type trend as the urban population increased. In addition to the above observations, Yang et al. [60] and Gurjar et al. [59] revealed that the relationship between PM_2.5_ concentrations and urbanization differed in various countries and megacities. One study reported the PM_2.5_ variations in urban and rural areas of eastern China between 2001 and 2015. According to this study’s findings, in 15-year averages, urban areas had greater concentrations of PM_2.5_ (about 61.0 μg/m^3^) than rural regions (about 52.7 μg/m^3^) [61]. The yearly average concentrations of PM2.5 in underdeveloped countries increased from 25.71 μg/m^3^ to 26.42 μg/m^3^, remaining at a higher level. From 1998 to 2014, urbanization overgrew from 26.53 to 33.1 percent [53].

## 3. PM Pollution: Sources, Elemental Composition, and Impacts

### 3.1. Sources of PM Pollution

PM is described as a solid or liquid that is suspended in the atmosphere and is often referred to as an aerosol. Such aerosols include fly ash, dust, soot, fumes, smoke, mists, and condensing vapors [62]. PM is generated as an outcome of both anthropogenic and non-anthropogenic activities, which shows time, seasonal, and locational variations [63]. Seinfeld and Pandis [64] list several earthly phenomena that are under the category of natural (non-anthropogenic or biogenic) processes, including sea sprays (which contain the elements Na, Br, and Cl), biological aerosols, volcanic eruptions, uncontrollable forest fires, and wind-borne dust from soil erosion (which includes the elements Al, Si, Ti, Ca, Mg, Fe, and Sr) [64,65].

Most of a sizable portion (fine and ultrafine) of PM sources is produced by various human (anthropogenic) activities and is released into the atmosphere primarily from traffic, agricultural operations, construction, demolition projects, road dust and other forms of transportation, and industrial sources such as electricity production, mining, welding, and building [66,67]. Any fuel that burns produces PM, including wood, gas [68], diesel from crude oil, and gasoline [69].

- Natural sources:
  - Windblown dust is mainly fugitive dust, often carried by the wind, observed mainly in arid and semi-arid regions, and contributes to PM_10_ [63].
  - Sea salt aerosols (diameter of less than one to few micrometers) originate from wind pressure at the ocean surface due to bursting of bubbles, jet drops, etc. Ali et al. [63] reported that sea salt spray contributes to 80 percent of the total PM concentration at seashores.
  - Volcanic particles (depending on types of magma, style of eruption, severity, temperature, pressure, and eruption’s duration) can make transient peaks in PM. These particles can transmit up to thousands of kilometers in the environment [70].
  - Wildfires are prevalent in grasslands, shrub lands, and forests in the summertime and significantly contribute to PM [71].
  - Biological processes generate‘primary biological aerosols’ such as plant debris, pollen grains, spores of bacteria, and fungus. They are dispersed into the air without going through any chemical changes [63].
- Anthropogenic sources:
  - Non-exhaust emission: Resuspended road dust and road wear particles accumulate on the surface, and brake and tire wear particles (mostly fine and coarse particles) are major contributors to non-exhaust emissions [63].
  - Brake wear emissions: Brake wear particles that consist of the lining of brakes and disk abrasion due to grinding, evaporation, and condensation of brake pad material generate PM less than 10 μm in diameter as well as potential toxic elements (PTEs) [72].
  - Exhaust emission sources: Exhaust PM emits from combustion and mainly arises as a consequence of partial lube oil and burned fuel, ash of fuel oil, sulfate, and vehicle exhausts’ agglomeration of tiny particles [73].
  - Industrial emissions: The major industrial PM emission sources are fuel combustion (oil, coal, and coke), gas turbines, and furnaces. PM can also be generated by the mechanical treatment of raw ingredients and cast operations. Fuel has high ash content and has significant potential to emit PM. Industrial emissions are the second largest contributor of PM in European regions [71].

### 3.2. Size Distribution and Elemental Composition of PM

PM composed of inorganic and organic solids and liquids particles differs in origin, shape, size, and composition [74]. According to their size, PM is classified by the WHO as coarse (PM_10_), fine (PM_2.5_), and ultrafine (PM_0.1_), having aerodynamic diameters of less than 10, 2.5, and 0.1 μm, respectively [35]. Total suspended particles (TSP) (diameter size <~100 nm) can further be classified as primary and secondary particles [63].

The term “primary PM” refers to particles released directly into the atmosphere [75]. The primary source of PM is traffic, mainly caused by brake wear and tire wear particles as well as the resuspended road dust [76]. At the same time, brake and tire wear particles may consist of heavy metals such as antimony (Sb), lead (Pb), cadmium (Cd), copper (Cu), and zinc (Zn) [77]. In addition, the crustal inorganic particles generated by pavement abrasion are frequently rich in minerals containing aluminum (Al), silicon (Si), sodium (Na), potassium (K), and calcium (Ca) [78]. Due to the small size, fine PM tend to float in the atmosphere for very lengthy time-spans (weeks, months, or years) and can travel long-range (hundreds or thousands of kilometers) [79]. As a result, the concentrations of various PM fractions can vary significantly from day to day if there are variations in atmospheric stability and wind occurrence patterns [79].

The term “secondary PM” refers to particles created in the atmosphere through gas-to-particle conversion processes [64]. The principal components of aerosols include metals, soot, salt particles, pollen, and spores. On the other hand, gases, including sulphates (SOx), volatile organic compounds (VOCs), nitrates (NOx), and ammonia, create secondary aerosols [62]. These procedures, which can change a particle’s makeup or enlarge it, proceed in three phases. The formation of the first nuclei or particles in the atmosphere occurs in the nucleation mode [80], which is dependent on gas concentration, temperature, and humidity in the atmosphere [81], as well as the conversion of the gaseous phase to a liquid or solid by condensation or chemical reaction [62]. Primary aerosols are created in the second stage, which is the condensation of heated gases. This event occurs similarly to the nucleation reaction [82]. Coagulation is the last stage in aerosol generation. Whole aerosols made in earlier processes may start to clump together due to Brownian motion [8] or turbulence and particle interaction [83]. As a result, particles expand in aerodynamic size, creating secondary particles from primary particles [84].

Coarse particles mainly consist of the Earth’s mineral crust, sea salt, biogenic substances, etc., while fine PM consist of aggregates associated with carbons, metal, and organic pollutants [85]. PM emitting sources and combustion factors are the key elements that govern PM chemical composition [63]. PM is generated as a consequence of a significant amount (80–90%) organic carbon and elemental carbon (EC, BC) combustion [86].

Inorganic ions are frequently detected as chemical components in PM (e.g., nitrates, sulfates, sodium, ammonium, magnesium, calcium, potassium, and chloride) and these are generally identified by ion chromatography columns. However, its composition may be further broadened to encompass all types of extremely astonishing and heterogeneous chemical mixtures, including VOCs, PAH, PTEs, crustal material, particle-bound water, and inorganic carbon [85]. Various types of metal elements are found in PM_10_ aerosols, largely produced by different emitting sources such as the material resuspension of the Earth’s crust (Ca, Al, Fe, Mg, K, Ti, etc.), industrial activities (Co, Fe, Cu, Cd, V, Cr, As, Ni, Mn, Ca, Zn, Sn, etc.), biomass (wood) burning (Mn, K, Pb, Cu, Na, Zn, etc.), coal combustion (As, Se, K, Cd, Zn, Mn, Na, Pb, Ca, Cu, Cr, Tl, etc.), vehicular emissions (Mn, Sb, Zn, Ba, Fe, Cu, Ca, V, Sn, Tl, Cr, Cd, Ni, Mg, and Pb), oil combustion (Ni and V), cement plants (Zn, Ca, Mn, Fe, Pb, Sb, Tl, Cd, Cu, As, Ni, etc.), and others, which are detected by inductively coupled plasma with atomic emission spectroscopy (ICP-AES) [87] and inductively coupled plasma optical emission spectroscopy (ICP-OES) [88]. On the other hand, Fourier transform infrared spectroscopy (FTIR), and X-ray diffraction (XRD) are used to identify minerals [89]. Field emission electron microscopy (FE-SEM) combined with energy-dispersive X-ray (EDX) are also used to analyze these elemental compositions and morphologies [89].

Additionally, the environmental relevance of endotoxins (enzymatic and sugar) and biological elements (such as allergens and microbial chemicals) hasalso been recognized in terms of PM, and have potential health risks. To identify particular markers of sugar (glucose, levoglucosan, and mannitol) in biogenic PM, high-performance anion-exchange chromatography with pulsed amperometric detection (HPAEC-PAD) is utilized [90]. Ultra-fine particles have a large abundance of PAHs, while particles (diameter less than 50 nm) mainly have a semi-volatile organic composition including hopanes and organic acids [91,92]. Incomplete combustion of organic material at high temperatures contributes to the formation of PAHs, while particles are emitted from different emitting sources such as coal and biomass combustion, traffic, and industrial sources [93]. According to Biswas et al. [94], PM_2.5_ and PM_10_ reach their highest concentration during the post-monsoon season. Another study by Zapletal et al. [87] reported that the daily average PM_10_ and PAHs concentration showed seasonal variation; the concentration is lower in the monsoon season and relatively higher in the pre-monsoon season. They also reported that the average daily PAHs concentration in Nepal (Tulsipur and Charikot) was 23.8 ng m^−3^ throughout the pre-monsoon season and 2.30 ng m^−3^ in the monsoon season. Hopane’s daily average concentration was 1.40 ng m^−3^ during the pre-monsoon in Tulsipur (Nepal) and 0.70 ng m^−3^ in Charikot (Nepal). However, basic available information on atmospheric PM_10_ and its constituents, such as elements and PAHs in the Mountains of Himalayan region, is still limited [95]. PM shows a stronger association with organic chemical substances such as ketones, quinones, olefins, aldehydes, and nitro-compounds [63].

### 3.3. Impacts of PM

#### 3.3.1. Ecological Impacts

Stresses that are both acute and chronic can be caused by disturbances brought on by the release of harmful compounds into the land, atmosphere, and water. After the stress is reduced, a successional process may eventually allow the ecosystem to regain some of its previous structure. For example, acute air pollution strains are typically short-lived (a day on average), with immediate impacts. On the contrary, chronic stressors are long-term (one-year average) stresses that impact the structure of several ecosystem levels and only become apparent after repeated exposures [11]. The following are some potential effects of PM pollutants on ecosystems: (i) pollutants accumulation in plants and other ecosystem components (viz. soil, surface water, and groundwater); (ii) harm to consumers as a result of pollutant accumulation; (iii) shifts in biodiversity due to changes in competition; (iv) biogeochemical cycles disruption; (v) disruption of stabilization and reduction in the capacity for self-regulation; and (vi) abolishment of stands and associations. These consequences, which impact ecosystem structure and operation, can be brought on by PM deposition, as covered in the following text [25].

Particles transferred to foliar surfaces from the atmosphere may stay on the leaf, bark, or twig surface for a long time, be absorbed via the leaf surface, or be expelled from the plant by re-suspending into the atmosphere [96]. There are three main ways that particles are deposited in the atmosphere and reach ecosystems:

1. Wet deposition, in which particles are settled in snow and rain;
2. The dry deposition is significantly slower;
3. The occult deposition is caused by cloud water, fog, and mist interception.

The impact of any PM deposited on above-ground plant components may be chemical or physical. The “inert” PM’s impacts are primarily physical, while hazardous particles have both chemical and physical effects [97].

Studies have yet to consider how particles affect people, groups, and ecosystems. Tolerant individuals have been chosen for tolerance at both the seedling and adult stages when exposed to trace metal or nitrate deposition [98]. They are found at low frequencies in populations while growing in unpolluted environments. Studies on PM deposition impacts, particularly those caused by chemically active and inert dust clouds, have shown that tolerant individuals in a plant demonstrate a broad spectrum of sensitivity, which is the foundation for the ‘natural selection’ of tolerant individuals [99]. At locations with significant nitrate and trace element deposition, the rapid evolution of some populations of tolerant species has been noted. A forest ecosystem may suffer from chronic pollution damage if sensitive species are lost, the tree canopy is reduced, or a residual layer of pollutant-tolerant herbs and shrubs—known as successional species—is maintained. A decrease in photosynthesis, crust development on leaves, early leaf fall, and leaf tissue loss were all caused by the dominating plants’ slower growth [100]. The growth of the dominating trees varied in response to variations in community makeup. The development of *Liriodendron tulipifera* L., *Cornusflorida* L., *Ostrya virginiana* (Mill.) K.Koch, and *Viburnum prunifolium* L. was boosted, likely as a result of less competition from more vulnerable species. At the same time, *Acer saccharum* Marshall was more prevalent in dusty sites at all strata levels. The growth of Rhododendron (*Rhododendron maximum* L.) and other conifers was impeded, probably due to the soil’s and leaf surfaces’ apparent alkalinization.

Cement dust releases calcium hydroxide on hydration, sometimes elevating leaf surface alkalinity to pH 12. This degree of alkalinity can penetrate the cuticle, hydrolyze wax and lipid components, and denature proteins, finally plasmolyzing the leaf cells. Cement kiln treatment for a brief period (two to three days) produced dose-specific response curves between the rate of dust exposure and net photosynthetic inhibition or foliar damage [10]. The leaves were repeatedly misted throughout the trial, but no long-lasting crust formed. Whether applied experimentally or in a contaminated field setting, alkaline dust containing significant amounts of MgO disturbed the outer epicuticular waxes on *Picea abies* (L.) H.Karst. needles [101].

Marine aerosol, which enters the air from the oceans after introducing air into the water column and bursting bubbles, can damage plant surfaces because it is frequently present around the surf line and therefore is in close proximity to potentially sensitive terrestrial receptors [102]. In coastal areas, the buildup of airborne salt particles on the leaves causes foliar damage and the extinction of plant species that cannot tolerate salt spray [103].

There have been reports of PM’s effects on phyllosphere-dwelling microbes [104]. After litterfall, decomposition is greatly aided by microbes, arthropods, insects living on the tree’s leaves, and other flora [105]. As a result, the decomposer community is weakened and the decomposition process after leaf fall is slowed down [106]. Early on, there is a significant influences on the decomposition of iron (Fe), Cu, Zn, chromium (Cr), nickel (Ni), and Pb-containing oak leaves [107]. Litter and dirt contain substantially fewer fungus mycelia [108]. The buildup of mineral nutrients and carbohydrates in dense, additional, slowly decaying litter impacts the ecosystem’s ability to absorb nutrients. Because of their dietary dependency on, and extended exposure to, particle deposition, epiphytic lichen, and Sphagnum moss plant communities are already threatened by PM exposure [109]. The health of the rhizosphere’s biota and the nutrients cycling required for plant development and vigor can be impacted by indirect PM impacts on plants that happen due to the soil [110]. The nitrogen and sulphur cycles, crucial to bacteria, make these components bio-available for plant absorption and development. In addition, direct fungi are necessary for plant development. They form mycorrhizae, mutualistic, symbiotic interactions essential to the intake of mineral nutrients, and are drawn to the roots by the exudates. The impacts of PM (especially nitrates, sulphates, and metals) on the development of the microorganisms engaged in nutrient cycling dictate how these pollutants affect ecosystems [25]. Acidification’s effects complicate the impacts of PM, including heavy metal contamination of mycorrhizal fungus communities [111]. As pH drops, heavy metals that have already been deposited and soil-borne Alcan can be mobilized and become more bio-available. Even lead deposition, nevertheless, has the potential to boost other, more resistant genotypes while decreasing other mycorrhizal species [94]. As a result, fungal population density, structure, and diversity can be less impacted by deposition, yet species composition can change. Other times, practical applications of Cd or Zn did cause decreases in the mycorrhizal fungi density [111,112].

Soil acidity is linked to both N and S deposition. PM effects via the soil integrate some of these element effects [113]. This suggests that NH_3_/NH_4_^+^ deposition causes changes to heath land through two mechanisms: (a) soil acidification and the loss of the cations, K^+^, Ca^2+^, and Mg^2+^, and (b) nitrogen enrichment, which causes “abnormal” plant growth rates and changed competitive dynamics [114].

Suspended PM mass concentrations, base cations, particle heavy metals, sulphate aerosol, and clouds of caustic compounds are likely the most significant indicators of the PM effect [115]. All of them are close to point sources and affect the turbidity of the atmosphere [116]. The impact of PM’s many components on the temperature distribution and radiation balance in the atmosphere varies [12]. However, vegetation has a considerable impact on PAR. Both sulphate and suspended dirt decrease PAR activity when present directly, whereas sulphate aerosol clouds significantly diminish PAR activity [13]. Surface solar visible radiation is thought to be reduced by 7–18% by regional haze. Therefore, the productivity of natural ecosystems and the output of crops may be impacted by the regional haze’s attenuation of PAR [115].

#### 3.3.2. Human

Particles with a diameter of fewer than 10 µm are known to have the most significant influence on human health. In human airways, size is significant because it determines the location of deposition in the lung [117]. Combining inertial impaction, gravity sedimentation, and Brownian diffusion, aerosol deposition in the human lung occurs [117]. Different PM sizes may be detected in the atmosphere, such as the PM_10–2.5_ that can enter the upper airways [118] and is deposited by a sedimentation or impaction process [117]. Through sedimentation and Brownian diffusion processes, PM_2.5_ is deposited in the lung, particularly in the alveoli; nevertheless, it may also enter the systemic circulation [118]. *Brownian diffusion* is the primary mechanism by which PM1 is deposited in the lung [117]. However, these particles can move from lung locations through systemic circulation [119] to the liver, heart, spleen, and brain [120]. However, they can also go from the olfactory bulb to the brain via a trans-synaptic pathway [120].

Based on its nature, the complex mixture of PM can cause distinct alterations in the tissues, which contain a water-soluble or a water-insoluble component [121]. According to Falcon-Rodriguez et al. [75], the water-soluble fraction can cause cell signaling, the release of inflammatory mediators, and oxidative stress, which damages DNA through a transition metal-dependent OH generation and suggests that H_2_O_2_ plays a significant role [122]. Furthermore, in vitro tests on the BEAS-2B cell line showed that exposure to the water-soluble fraction resulted in more significant oxidant generation, inflammatory cytokine concentration, and IL-8 synthesis than exposure to the insoluble fraction. Similarly, neutrophil invasion and lavage protein concentrations in rats are increased by intratracheal instillation of water-soluble and insoluble fractions. However, after exposure to the water-soluble fraction, neutrophil and protein increases were higher [75]. Additionally, water-soluble and insoluble organic aerosols significantly enhance the oxidative characteristics of ambient PM [123].

Numerous investigations dating back to 1980 have noted that exposure to PM causes more cancer cases and fatalities. Asthma, fibrosis, and chronic obstructive pulmonary disease (COPD) are lung illnesses that are known to be brought on by exposure to PM [124]. In addition, increased perivascular and peribronchiolar inflammation follows exposure to diesel exhaust particles [125].

Due to their organic or inorganic composition [126], exposure to fine or ultrafine particles causes ROS-mediated oxidative stress and changes the permeability of epithelial cells [127]. The hydroxyl radical produced by hydrogen peroxide after exposure to PM is a primary kind of ROS [128]. Additionally, PM_2.5_ can generate superoxide, forming hydrogen peroxide [129]. The primary free radical in the lungs, H_2_O_2_, can cause oxidative stress, which can harm cells [75]. The International Agency for Research on Cancer recently categorized outdoor air pollution as a group-I carcinogen [130]. Metals and PAHs are the two main elements in particles that contribute to oxidative stress. Both are potent mutagenic and carcinogenic agents [75]. About 7% of all deaths in 2010 that may be attributed to PM_2.5_ were explicitly caused by malignancies of the trachea, bronchi, or lungs [131]. According to certain research, exposure to PM can cause lung cancer in non-smokers [132]; nonetheless, smokers are more likely to acquire lung cancer [75].

## 4. Green Urban Architecture and Their Impact

GI methods have been widely adopted in several metropolitan regions worldwide for development to introduce urban greening concepts to lessen the effects of dangerous pollutants in the atmosphere [133]. GI practices have been shown to be effective in lowering the harmful PM pollutants, including trees, shrubs, lawns, urban farming, vertical green, living walls [134], urban forests, green façade, urban greenery, vegetation or green barriers, street canyons, and green walls (GWs) (Table 1) [135]. In various locations across the world, GI techniques have shown promise in reducing air pollution in urban areas. In order to reduce PM pollution, GI methods are commonly utilized in the United States, Australia, and Europe [133]. The 2030 EU Biodiversity Strategy [136] and UN-Habitat [135] provided and supported the sustainable and equitable development of urban areas, highlighting the need for GI in cities.

Numerous studies have reported on the impact of urban greenery on PM reduction levels. However, most of them emphasize the part that trees play in this phenomenon, despite the fact that herbaceous plants greatly enhance the ability of trees to catch the light [146]. According to McPherson et al. [19], Chicago, Illinois, has an annual accumulation of 212 t per year of PM_10_ in trees. Compared to external forests, urban plants have been shown to lower PM concentrations by 9.1% in Shanghai, China [147]. Urban plants’ ability to collect PM may also allow them to eliminate ambient airborne particles linked to heavy metals. According to studies, urban trees in the United States eliminated around 215,000 t of total airborne PM_10_ [18], while an increase in tree cover from 3.7% to 16.5% in the West Midlands removed about 200 t of PM_10_ annually [97]. Additionally, the canopies of a central Japanese coniferous forest and a Norway spruce forest dramatically changed the sulphur content and sedimentation rate of PM_2.5_ [148,149]. Ninety-six tons of air pollutants were eliminated in Scotlandville, Louisiana, U.S., within forest canopy coverage of 23.7 percent [150]. Barcelona, Spain, had a total annual pollution removal of 305 t per year. In contrast, Brooklyn Industrial Precinct in Perth, Australia’s western suburbs had a yearly pollutant removal of 294 t [133]. In Shanghai, China, the annual PM_2.5_ reduction can reach 442.4 t per year [151]. There may be a connection between trees’ ability to purify the air and the following factors: an increase in vegetation cover lowers the sources of PM_2.5_; various tree organs can absorb PM; a decrease in wind speed may cause PM fallout; and a change in wind direction may stop PM_2.5_ from being transported into specific areas [152]. The capacity of trees to filter out PM_2.5_ is influenced by many variables, including meteorological conditions, tree biological features, and atmospheric PM_2.5_ and PM_10_ concentrations [152].

### 4.1. Urban Meadows

Urban meadows are also a key component of cities’ nature-based strategies for trapping PM released from the street (or transport) because of species biodiversity (plants with various growth and development patterns, stem and leaf morphology) and the height of canopies, particularly in areas where shrubs and trees are impossible and undesirable [153]. In order to maximize the effectiveness of PM immobilization effects, vegetation should be located as close to the emission source as feasible [153,154,155]. The effectiveness of PM accumulation through plants is reduced if the vegetation is far from the sources. Even trees 60 m from a gravel road and 10 m tall had no discernible impact on air PM_10_ concentrations [156]. Therefore, it is reasonable to assume that roadside meadows will significantly reduce the amount of PM in the ambient air near roadways [157]. Sadly, there needs to be more information in the literature about how effective meadow plants are at cleaning the air around us. Sixteen types of herbaceous plants (grasses and forbs) were shown by Weber et al. [146] to be able to acquire PM from road sources. Przybysz et al. [141] recently reported perennial meadows plants (*Centaurea scabiosa* L., *Echium vulgare* L., and *Convolvulus arvensis* L.) species accumulate more PM and are better adapted than annual meadows plants species (such as *Chenopodium album* L., *Achillea millefolium* L., and *Echium vulgare* L.). The ability of the plants to withstand urban-specific growth circumstances, such as poor soil quality, heat, drought, salt stress, and air pollution, should be considered when choosing plants for the urban meadows and targeted for PM accumulation [158,159,160,161]. Therefore, urban meadows can be a crucial strategy to purify the air in polluted and populated cities.

### 4.2. Green Roofs

Greening horizontal systems with extensive and intensive GRs are among the most common technologies used widely nowadays. This is mandated by many international laws and policies, especially in northern Europe. Many researchers looked into their economic advantages [142]. For instance, the capacity of *Sedum album* L. to trap PM was 0.42 g m^−2^ y^−1^, compared to *Agrostis stolonifera* L. and *Festuca rubra* L. with the potentials of 1.81 and 3.21 g m^−2^ y^−1^, respectively. Other *Sedum* species (*S. palmeri* S. Watson and *S. reflexum* L. have lower efficacy) and plants such as *Pittosporum tobira* (Thunb.) W.T. Aiton (1.38 μg cm^−2^ h^−1^) and *Erigeron karvinskianus* (DC.) Kuntze (1.62 μg cm^−2^ h^−1^) had lower PM deposition rates in semiarid regions but this species had a higher rate, reaching 29.32 μg cm^−2^ h^−1^ [162]. Due to the potential for more numerous and varied plants, intensive and semi-intensive GRs are more effective at reducing PM deposition. In a Montreal study, the *Pinus mugo* var. *pumilio* (Haenke) Zenari covered GRs on the wood-heated buildings were able to remove 4 g m^−2^ of PM_10_ and 1.52 g m^−2^ of PM_2.5_ yearly [163]. GRs are less effective than trees. However, occasionally, they can catch PM at levels comparable to trees. It is essential to note that GRs work as a supplement to trees rather than as a rival to them [135].

### 4.3. Vertical Greening Systems

Vertical greening systems can be divided into green façades and GWs systems according to their rising method [142].

#### 4.3.1. Green Walls

GWs have recently made significant progress in being used for PM collection. GWs also benefit a building’s acoustics since it lowers ambient noise levels [164]. GW systems are also known as living walls and vertical gardens. Active mechanical ventilation supports air movement through the canopy, the growth medium, and the plant rhizosphere to boost plants’ purifying capacity. Weerakkody et al. [134] also explored the role of living wall systems in reducing PM pollution; PM capture was investigated at Birmingham New Street railway station. Active GW technology, also known as botanical filtration, is generally known for its effectiveness in PM and VOCs abatement indoors [165]. Giachetta and Magliocco [166] reported that this advantage is relatively minimal in the case of a thin-layer vegetation cover.

#### 4.3.2. Green Façade

The basis of a green façade is the usage of soil-bounded plants, generally herbaceous or woody climbers, that are either directly affixed to the surface of the building, as in traditional construction (direct green façade), or are supported by cables or trellises (indirect green façade) [167]. Ivy (Hedera helix), the superior green façade species now being researched, is the most prevalent species in direct green façades worldwide [142]. Green façades can increase the PM collection area of a building more than only GRs. For instance, greening a cubic building’s façade covers an area four times that of the roof [168].

### 4.4. Urban Agriculture

Urban agriculture and horticulture are other trends in GI. Shortening of the food chain is a reaction to the rising market demand for fresh and regional foods. It fits in wonderfully with a circular city, where wastewater and organic waste are recycled. It incorporates various cutting-edge environmentally friendly technology, including hydro- and aeroponics, vertical farming, rooftop gardens, and more conventional methods of food production, including allotments and individual kitchen gardens. Community gardens are a relatively new but steadily growing method of food production that aims to unite local communities. Moreover, urban food production provides food and addresses PM pollution [135].

## 5. Avenue Trees: Potentials and Possibilities

**Figure 3:**
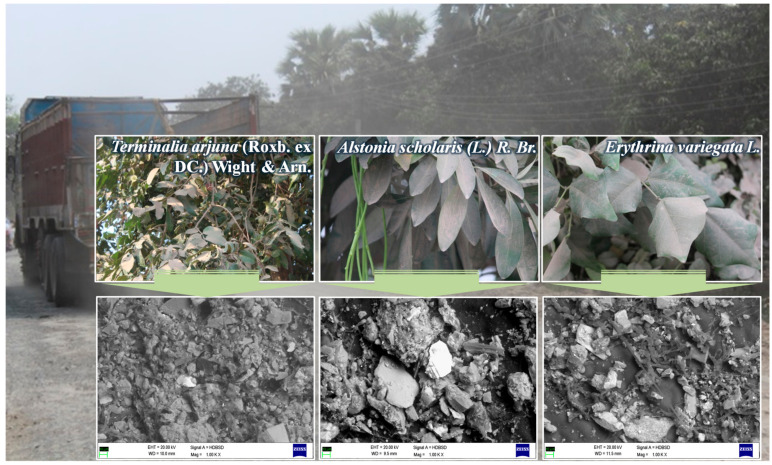
Plants are continuously exposed to heterogenous particulate pollution in urban agglomerations. Leaves are the primary place for the deposition of PM. Normal and scanning electron microscopic (magnification 1000×) images of selected plants with particulate deposition inMalda, West Bengal, India.

Even while rising PM concentrations cause clear physical harm, the high rate of economic expansion in metropolitan areas makes it impossible to totally stop PM generation from many sources. Therefore, a study must be carried out on ways to reduce the amounts of other atmospheric pollutants while also removing atmospheric PM (Figure 3). The possibility of using trees to reduce the amount of air particles has been the subject of research. Recent studies have shown that avenue trees, particularly in urban and suburban environments, may dramatically lower PM_2.5,_ and absorb gaseous air pollutants [152,169]. Jayasooriya et al. [133] reported that combining various GI, such as GWs and GRs, did not significantly enhance the air quality. However, it did have more immediate advantages, including reduced building energy use. Among the various GI, trees had the best potential to remove air pollutants [133]. Avenue trees are, therefore, environmentally friendly ways to drastically lower atmospheric PM because trees have more significant leaf surface areas than shrubs and herbs, which improves their PM uptake efficiency [97]. Because they frequently have substantial, massive structures, trees can cause air turbulence, which increases the buildup of PM on their leaf surfaces [170,171]. In contrast to other surfaces in similar conditions, the large surface areas of the leaves of urban plants have greater efficiency for collecting airborne PM [172,173]. Compared to species with smoother blade-like surfaces, broadleaved species have more excellent PM capture capabilities [174]. Furthermore, because they may constantly absorb PM, evergreen broadleaf species have significant consequences for air purification. Diverse plant species have different capacities for purifying PM at the level of a single leaf because they have unique leaf shapes and morphological characteristics (such as ravines, stomata, and epidermal trichomes) that efficiently take PM from the atmosphere [175]. The size of leaves may significantly affect the amount of PM that accumulates, with complex leaf forms (such as lobed leaves) showing a larger capacity for PM capture than simple leaf designs [134].

According to the findings of an analysis of PM deposited on the leaves of different *Ficus* species (*F. benghalensis* L., *F. microcarpa* L., *F. religiosa* L., and others) with similar leaf structures (smoother surfaces and hairless), the PM loading on leaves was significantly different in different areas. This may be connected to variations in atmospheric PM levels [176,177]. According to some research, different PM concentrations may be connected to particle diameter. Therefore, the leaf anatomy and morphology properties were tightly connected to the PM deposition [178]. According to Han et al. [10], the leafy appendages, such as hair-like structures trichomes, and others, enhance the roughness of the surface and areas of PM interception as well as reduce the PM removal quantity by wind [179,180]. Additionally, less PM is accumulated on leaf surfaces due to waxy epicuticles’ hydrophobicity [10]. According to Popek et al. [181], cuticular leaf waxes would act as a restricting barrier for water-soluble materials due to their hydrophobic nature. According to studies, leaves with a lanceolate form retain more PM than leaves with other shapes (e.g., elliptic, obovate, linear-shaped and needlelike) [10]. However, compared to species with more extensive leaf areas and longer petioles, those with smaller leaf surface areas and smaller petioles acquired more PM on their leaves [182]. Even though the majority of surfaces are coated with wax, leaves can still absorb particulates through the stomatal pathways [183]. Therefore, increased levels of leaf stomata may have the ability to trap ultrafine PM on leaf surface areas. PM, however, has the potential to harm epicuticular waxes and affect stomata function [10]. Previous research demonstrated that evergreen species were better able to minimize PM than deciduous trees since their leaves remained on the tree throughout the year, particularly in the winter and spring when hazy fog is more common [165]. According to earlier studies, the tree species listed in Table 2 effectively remove PM from the environment.

Modern wireless communication technologies and portable, inexpensive air pollution sensors allow the densification of existing networks of monitoring and capturing tempo-spatial air quality variations in urban areas [186]. For example, long-term assessment by strategically positioned sensors can show the variance in the air quality before and after vegetation plantation and monitor the immediate impact of vegetation on air purification [187]. Another crucial factor in determining the removal of air pollution is the greenery structure, which is most frequently defined primarily through the Leaf Area Index (LAI). The mapping of LAI temporal and spatial dynamics is challenging at higher scales due to the approach’s time- and labor-intensive nature and scaling issues. Moreover, general LAI rates for the entire urban forestry of a similar kind (such as broad-leaved/coniferous) are used without species metrics [138].

On the other hand, remote sensing approaches address these issues and provide broad and continuous geographical coverage for reproducible monitoring of vegetation phenology [188]. In order to evaluate the contribution of vegetation to air pollution abatement, some studies have previously employed remote sensing techniques depending on aerial “light detection and ranging” (LiDAR) [189] or aerial and satellite imaging [190,191]. Although airborne and aerospace-based passive remote sensing is perhaps practical for modeling the removal of air pollution in regions with a homogenous land area cover on a coarse scale, examining the intricate composition of each tree is beyond capacity. Conversely, airborne LiDAR enables accurate canopy structure assessment [192]. However, the expense of data collection can be too high for the local authorities. In order to balance costs and spatial resolution, passive sensors on board “unmanned aerial systems” (UAS) alleviate certain constraints of aerial LiDAR [193]. UAS optical aerial imagery methods utilizing the structure from motion (SfM) algorithm enable high-resolution evaluation of green space structures overseveral square kilometers, making it appropriate forevaluating the ecosystem services provided by urban green spaces such as public spaces and parks [93]. Despite the latest usesof UAS-SfM in urban forest inventories [93], this method still needs to be considered when modeling how urban greenery reduces PM pollution. Currently, the two primary models used to explore the PM removal capabilities of trees at the city-scale are the ‘CITY green model’ and the ‘i-Tree model’ [10,22]. These models primarily include meteorological parameters, air pollutants, and urban trees structures data to estimate dust removal.

While several studies have assessed the amount of pollution trees can remove (for instance, 18–21), most of this research does not explicitly relate the removal of pollutants to enhanced human health-related impacts and associated health values. One studyin London, England, that connected PM removal to health consequences projected that 10 × 10 km grids with 25 percent tree cover would be effective at removing approximately 91 t of PM_10_ yearly, equivalent to preventing two mortalities and two hospitalizations per year [194]. According to Nowak et al. [195], the annual PM_2.5_ removal efficiency of trees in ten U.S. cities in 2010 ranged from 4.7 t to 64.5 t in Syracuse and Atlanta, respectively. The estimated yearly costs for the improvements in mortality, hospitalization, and respiratory problems brought on by the reduction of PM_2.5_ in these areas varied from $1.1 to $60.1 million in Syracuse and New York City (NYC), respectively. The average annual mortality saved in each city was about one person, although it might reach 7.6 people annually in NYC. In 2010, 17.4 million tons of air pollution in the entire U.S. were removed by trees and forests, at a cost to human health of 6.8 billion U.S. $ [196,197]. Rural regions had reduced most of the pollution, whereas urbanized regions had the majority of health effects and benefits. More than 850 human deaths and 670,000 cases of acute respiratory disease were prevented, positively influencing health [139].

Avenue trees can be used as biomonitoring contaminants in urban environments, and are the best choice for eco-friendly and cost-effective plant components. Additionally, they cause no secondary pollution, are simple to collect, and can be studied inexpensively [175,198]. As a result, plants may be effectively used to remove airborne pollutants in urban settings. However, more information is needed about the variations in PM capture efficiency across different kinds of urban greening plants. When choosing the best plant species for urban greening, the capacity to trap PM is crucial [199]. Furthermore, to maximize the advantages of these plants in varied urban areas, it is crucial to understand how well different plant species can filter airborne PM contaminants [200].

## 6. Conclusions and Future Prospects

Diverse plant species provide a rich supply of PM pollution reduction through their canopies in urban areas because of their various defensive mechanisms and structural and gas-exchange features. In addition, GIs emphasize the potential for trees and their biodiversity value. According to a rating system for 100 regularly utilized tree species based on their ability to filter out PM_2.5_, many conifers perform optimally due to their year-round foliage, thick, fine-textured canopies, and high leaf area index [201]. Regretfully, not all widely spread urban tree species performed as effectively as they could have. Nevertheless, there are still many things that could be improved in our understanding of this phenomenon. The main issue is the need for consistent scales for the number of pollutants absorbed, making it challenging to compare the best species for reducing air pollution. However, the key characteristics for increased pollutant deposition and infiltration are well understood. They include the long in-leaf season, ideal (moderate) canopy density and porosity, small size and complexity of leaves (needles), rough leaf surfaces (including grooves and trichomes), and high epicuticular wax content [202]. We are also still determining those characteristics of leaves that enhance PM capture. However, the significance of stomatal density, size, and the quality of surface waxes, as well as new prospective plant and tree species, still requires further study, despite there being consensus on the advantages of GI for urbanization, which is predicted to lower ambient PM_10_ by 26% locally [97]. Additionally, green areas significantly benefit the environment (including CO_2_ sequestration), biodiversity, and human health and wellness [154]. Incorporating various forms of GI is essential to maximizing plants’ ability to catch PM [203], which results in a noticeable increase in leaf area index and capture capacity. However, there is still an opportunity for advancement in the use of plants to reduce PM. The ability of a species to reduce PM under local climatic circumstances and its resistance to stressors and VOC emissions should be considered while choosing the best species for the purpose.

## Figures and Tables

**Table 1:** Different types of GIs.

Study Site	GI System	Name of Plant	Size Fractions of PM Pollutants	References
Birmingham New Street railway station	Living wall systems	*Hebe albicans* Cockayne, *Buxus sempervirens* L., *Hebe x youngii*, and *Thymus vulgaris* L.	PM_1_, PM_2.5_ and PM_10_	[134]
National Institute of Social Insurance (INPS) Green Facade, Italy	Vertical greening system	*Hedera helix* Lowe, Cistus ‘Jessamy Beauty’, *Trachelospermum jasminoides* (Lindl.) Lem., *Phlomis fruticosa* L.	PM_2.5_ and PM_10_	[137]
Brooklyn industrial precinct, Melbourne, Australia	Tree	*Eucalyptus cladocalyx* F.Muell.	PM_2.5_ and PM_10_	[133]
	Green Roof (GR)	*Eucalyptus macrocarpa* Hook.		
	GW	*Laurus nobilis* L.		
Ghent, Belgium	Tree crowns and an urban street canyon	*Platanus acerifolia* (Aiton) Willd.	PM (not specific)	[7]
Ostrava–Radvanice, Czechia	Urban greenery	*Acer pseudoplatanus* L., *Salix daphnoides* Vill.	PM_10_	[138]
Canada	Urban forests	Trees	PM_2.5_	[139]
New York City	Urban roof top vegetable farm	Vegetables	PM_2.5_	[140]
Warsaw, Poland	Urban meadows	*Chenopodium album* L. *Achillea millefolium* L., *Echium vulgare* L., *Centaurea scabiosa* L., *Echium vulgare* L., and *Convolvulus arvensis* L.	PM (not specific)	[141]
Genoa, Italy	Green façade	*Hedera helix* Lowe	_-_	[142]
	Living wall system	Evergreen climbing plants and small shrubs		
Santiago, Chile	GRs and GWs	*Sedum album* L.	PM_2.5_	[143]
Sheffield, UK	Green barriers	*Thuja occidentalis* L., *Hedera helix* Lowe, *Phyllostachys nigra* (Lodd. ex Lindl.) Munro	PM_1_ and PM_2.5_	[144]
Beijing, China	Urban Forest Park	Trees/shrubs (lawn grass flower, coniferous broadleaved mixed)	PM_10_, PM_2.5_ and PM_1.0_	[145]

**Table 2:** List of PM load capacity on different plant species from previous studies.

Study Site	Name of Plant	Family	Habitat	PM Load	Reference
Rourkela Steel Plant, Rourkela, India	*Albizia lebbeck* (L.) Benth.	Fabaceae	Evergreen	0.405 mg/cm^2^	[184]
Rourkela Steel Plant, Rourkela, India	*Alstonia scholaris* L.R.Br.	Apocynaceae	Evergreen	1.352 mg/cm^2^	[184]
Rourkela Steel Plant, Rourkela, India	*Anthocephalus indicus* A.Rich.	Rubiaceae	Deciduous	0.743 mg/cm^2^	[184]
Rourkela Steel Plant, Rourkela, India	*Bougainvillea spectabilis* Wild.	Nyctaginaceae	Semi evergreen	0.437 mg/cm^2^	[184]
Rourkela Steel Plant, Rourkela, India	*Caesalpinea pulcherima* (L.) SW.	Fabaceae	Semi evergreen	0.179 mg/cm^2^	[184]
Rourkela Steel Plant, Rourkela, India	*Cassia auriculata* L.	Fabaceae	Semi evergreen	0.546 mg/cm^2^	[184]
Rourkela Steel Plant, Rourkela, India	*Cassia siamea* Lam.	Fabaceae	Evergreen medium-sized	0.574 mg/cm^2^	[184]
Rourkela Steel Plant, Rourkela, India	*Delonix regia* (Bojer ex Hook.) Raf.	Fabaceae	Evergreen	0.137 mg/cm^2^	[184]
Rourkela Steel Plant, Rourkela, India	*Ficus religiosa* L.	Moraceae	Evergreen	0.493 mg/cm^2^	[184]
Rourkela Steel Plant, Rourkela, India	*Lagerstroemia speciosa* (L.) Pers.	Lythraceae	Evergreen medium-sized	1.310 mg/cm^2^	[184]
Rourkela Steel Plant, Rourkela, India	*Mimusops elengi* L.	Sapotaceae	Evergreen	0.652 mg/cm^2^	[184]
Rourkela Steel Plant, Rourkela, India	*Peltophorum inerme* (Roxb.) Navesex Fernandez Villar	Fabaceae	Deciduous	0.729 mg/cm^2^	[184]
Rourkela Steel Plant, Rourkela, India	*Swietenia mahagoni* (L.) Lacq.	Meliaceae	Evergreen	0.486 mg/cm^2^	[184]
Rourkela Steel Plant, Rourkela, India	*Tabebuia aurea* Benth Hook.f.ex S. Moore	Bignoniaceae	Deciduous medium sized	0.552 mg/cm^2^	[184]
Rourkela Steel Plant, Rourkela, India	*Thevetia nerifolia* Juss Ex. Steud	Apocynaceae	Evergreen	0.355 mg/cm^2^	[184]
Kunming City, China	*Magnolia grandiflora* L.	Magnoliaceae	Evergreen	4.20 g m^−2^	[175]
Kunming City, China	*Platanus acerifolia* Ait.	Platanaceae	Evergreen deciduous	3.43 g m^−2^	[175]
Kunming City, China	*Osmanthus fragrans* (Thunb.) Lour.	Oleaceae	Evergreen	2.25 g m^−2^	[175]
Kunming City, China	*Ligustrun lucidum* Ait	Oleaceae	Evergreen	1.47 g m^−2^	[175]
Kunming City, China	*Cinnamomum camphora* (L.) Presl.	Lauraceae	Evergreen	0.99 g m^−2^	[175]
Kunming City, China	*Cinnamomum japonicum* Sieb	Lauraceae	Evergreen	2.53 g m^−2^	[175]
Kunming City, China	*Photinia glomerata* Rehd. et Wils.	Rosaceae	Deciduous	1.83 g m^−2^	[175]
Kunming City, China	*Prunus majestica* Koehne	Rosaceae	Evergreen	1.34 g m^−2^	[175]
Kunming City, China	*Prunus cerasifera* f. atropurpurea	Rosaceae	Evergreen	1.6 g m^−2^	[175]
Kunming City, China	*Celtis kunmingensis* C.C.Cheng & D.Y.Hong	Ulmaceae	Deciduous	1.71 g m^−2^	[175]
Kunming City China	*Euonymus japonica* Thunb.	Celastraceae	Evergreen	1.9 g m^−2^	[175]
Kunming City, China	*Loropetalum chinense* var. rubrum	Hamamelidaceae	Evergreen	2.46 g m^−2^	[175]
Kunming City, China	*Rhododendron pulchrum* Sweet	Ericaceae	Semi evergreen	2.12 g m^−2^	[175]
Debrecen, Hungary	*Acer saccharinum* L.	Sapindaceae	Deciduous	13.9 g m^−2^	[185]
Debrecen, Hungary	*Tilia europaea* L.	Malvaceae	Deciduous	464 g m^−2^	[185]
Debrecen, Hungary	*Fraxinus excelsior* L.	Oleaceae	Deciduous	41.5 g m^−2^	[185]
Debrecen, Hungary	*Tilia platyphyllos* Scop.	Malvaceae	Deciduous	313.6 g m^−2^	[185]
Debrecen, Hungary	*Cydonia oblonga* Mill.	Rosaceae	Deciduous	254.6 g m^−2^	[185]
Debrecen, Hungary	*Elaeagnus angustifolia* L.	Elaeagnacea	Deciduous	215.9 g m^−2^	[185]
Debrecen, Hungary	*Ulmus pumila* L.	Ulmaceae	Deciduous	123.6 g m^−2^	[185]
Debrecen, Hungary	*Gleditsia triacanthos* L.	Legumes	Deciduous	89.2 g m^−2^	[185]
Debrecen, Hungary	*Picea pungens* Engelm.	Pinaceae	Coniferous evergreen	86.5 g m^−2^	[185]
Debrecen, Hungary	*Sorbus aucuparia* Poir.	Rosaceae	Evergreen	68.2 g m^−2^	[185]
Debrecen, Hungary	*Salix alba* L.	Salicaceae	Deciduous	64.4 g m^−2^	[185]
Jinju, Gyeongnam Province, Republic of Korea	*Pinus densiflora* Siebold & Zucc.	Pinaceae	Evergreen	24.6 µg cm^−2^	[186]
Jinju, Gyeongnam Province, Republic of Korea	*Quercus salicina* Blume	Fagaceae	Evergreen	47.4 µg cm^−2^	[186]
Jinju, Gyeongnam Province, Republic of Korea	*Quercus glauca* Thub.	Fagaceae	Evergreen	27.76 µg cm^−2^	[186]
Jinju, Gyeongnam Province, Republic of Korea	*Rhaphiolepis indica* (L.) Lindl. var. *umbellata* (Thunb. ex Murray) H.Ohashi	Rosaceae	Evergreen	22.94 µg cm^−2^	[186]
Jinju, Gyeongnam Province, Republic of Korea	*Illicium anisatum* L.	Illiciaceae	Evergreen	13.72 µg cm^−2^	[186]
Jinju, Gyeongnam Province, Republic of Korea	*Ginkgo biloba* L.	Ginkgoaceae	Evergreen	23.58 µg cm^−2^	[186]
Jinju, Gyeongnam Province, Republic of Korea	*Machilus thunbergia*Siebold and Zucc. ex Meisn.	Lauraceae	Evergreen	13.64 µg cm^−2^	[186]

## Data Availability

Data used in this review article were obtained from the databases of WHO (https://www.who.int/data/gho/data/themes/air-pollution/who-air-quality-database, accessed on 11 November 2022).
